# Prevention and Reversal of Diabetes by All-Trans Retinoid Acid and Exendin-4 in NOD Mice

**DOI:** 10.1155/2014/435481

**Published:** 2014-06-03

**Authors:** Jyuhn-Huarng Juang, Yang-Hau Van, Chien-Hung Kuo, Mei-Yin Lin, Ying-Hsiu Liu, Han-Ying Chang

**Affiliations:** ^1^Division of Endocrinology and Metabolism, Department of Internal Medicine, Chang Gung University and Chang Gung Memorial Hospital, 5 Fu-Shin Street, Kweishan, Taoyuan 333, Taiwan; ^2^Division of Pediatric Endocrinology, Department of Pediatrics, Chang Gung Memorial Hospital, 5 Fu-Shin Street, Kweishan, Taoyuan 333, Taiwan; ^3^Biomedical Technology and Device Research Laboratories, Industrial Technology Research Institute of Taiwan, 195 Sec. 4, Chung Hsing Road, Chutung, Hsinchu 31040, Taiwan

## Abstract

It has been shown that all-trans retinoid acid (ATRA) hinders the development of autoimmune diabetes by inducing immune tolerance status. Meanwhile, exendin-4 increases beta-cell function and mass. Thus, we hypothesized that ATRA and exendin-4 combination therapy would prevent and reverse autoimmune diabetes. NOD/scid mice were intravenously transferred with splenocytes isolated from 12-week-old female NOD mice. After adoptive transfer, mice were treated with vehicle, ATRA (0.5 mg/mouse intraperitoneally every other day), exendin-4 (3 **μ**g/kg subcutaneously twice daily), or combination for 6 weeks. Compared with vehicle, ATRA (*P* = 0.022) and ATRA plus exendin-4 (*P* = 0.013) treatment delayed the onset of diabetes. The pancreatic insulin content in mice treated with ATRA (*P* = 0.013) and exendin-4 (*P* < 0.02) was significantly higher than that of control mice. All but one spontaneous diabetic NOD mouse treated with ATRA and/or exendin-4 remained persistent hyperglycemic. ATRA and/or exendin-4 treatment did not alter their blood glucose levels and survival. Our results indicate that, before the onset of autoimmune diabetes, ATRA and exendin-4 treatment alone preserves pancreatic beta cells; ATRA and ATRA plus exendin-4 treatment delays the onset of autoimmune diabetes. However, after the onset of autoimmune diabetes, ATRA and/or exendin-4 treatment is unable to reverse hyperglycemia or improve survival.

## 1. Introduction

Type 1 diabetes is characterized by the progressive loss of pancreatic beta cells caused by autoimmune attack [1]. Although beta-cell mass is markedly diminished in long-standing type 1 diabetics, residual beta cells can be detected and new beta-cell formation may occur in these patients several decades after the disease onset [2]. This observation has led to researches to induce remission of diabetes by targeting beta-cell autoimmunity and regeneration.

All-trans retinoid acid (ATRA) is a potent vitamin A derivative that has been clinically used for the treatment of acute promyelocytic leukemia [3] and skin disease [4]. Recently, Van et al. demonstrated ATRA treatment inhibited diabetes in NOD mice by inducing Treg cell-dependent immune tolerance [5]. Exendin-4 (exenatide) is a glucagon-like peptide-1 (GLP-1) receptor agonist resistant to dipeptidyl peptidase-IV-mediated inactivation [6] which exhibits sustainable GLP-1 effects including stimulation of glucose-dependent insulin secretion and biosynthesis and suppression of glucagon secretion, gastric emptying, and appetite [7]. In addition, exendin-4 stimulates beta-cell growth and differentiation [8], inhibits beta-cell apoptosis [9], and delays the onset of diabetes [10]. Thus, we hypothesized that the combination therapy of ATRA and exendin-4 would inhibit autoimmune destruction and enhance the growth and function of pancreatic beta cells, therefore, prevent and reverse autoimmune diabetes.

## 2. Materials and Methods

### 2.1. Animals

NOD/scid (National Laboratory Animal Center and National Taiwan University, Taiwan) and NOD (National Defense Medical Center, Taiwan) mice were bred and housed in a specific pathogen-free environment in the animal facility at the Chang Gung Memorial Hospital. Blood was obtained from the snipped tail, and glucose was measured with a portable glucose meter (One Touch II, Lifescan Inc., Milpitas, CA, USA). Normoglycemia was defined as nonfasting blood glucose levels <200 mg/dL. All animal protocols were approved by the Ethics Committee of Chang Gung Memorial Hospital.

### 2.2. Adoptive Transfer

Splenocytes were isolated from 12-week-old female NOD mice. Nondepleted splenocytes (1 × 10^7^/mouse) were intravenously transferred into NOD/scid mice [5].

### 2.3. ATRA and Exendin-4 Treatment

NOD/scid mice with adoptive transfer and spontaneous diabetic NOD mice were treated with vehicle (as control), ATRA (Sigma, St. Louis, MO, 0.5 mg/mouse intraperitoneally every other day) [5], exendin-4 (Sigma, St. Louis, MO, 3 *μ*g/kg subcutaneously twice daily) [11], or combination for 6 weeks.

### 2.4. Immunohistochemistry of the Pancreas

The pancreases of NOD/scid mice were removed before death, fixed in formalin solution, and processed for paraffin embedding and sectioning. Sections of the pancreases were stained for the endocrine beta cells with a guinea pig antiswine insulin antibody (Dako Co., Glostrup, Denmark) [11], Pdx1, and Ki67 (Abcam, California, CA). The degree of insulitis was evaluated by the mononuclear cell infiltration around or in the islet.

### 2.5. Insulin Content of the Pancreas

The pancreases of NOD/scid mice were removed before death and homogenized in acid ethanol. After homogenization, the samples were extracted overnight at 4°C. On the following day, they were centrifuged at 2,400 rpm for 30 min, and the supernatant was stored at −20°C. The pellet was again homogenized in acid ethanol and left 2 h at 4°C. Above procedure was repeated and insulin was extracted overnight. After centrifugation, the supernatant was added to the previous extraction sample and kept in −20°C freezer till assay. Insulin was measured by radioimmunoassay with INSI-PR kit (CIS US Inc., USA) [11].

### 2.6. Statistical Analysis

Results were expressed as mean and standard deviation. Kaplan-Meier survival analysis was used to compare cumulative diabetes incidence. ANOVA were employed for comparisons among multiple groups. A value of *P* < 0.05 was considered significant.

## 3. Results

### 3.1. Prevention Study

**Figure 1:**
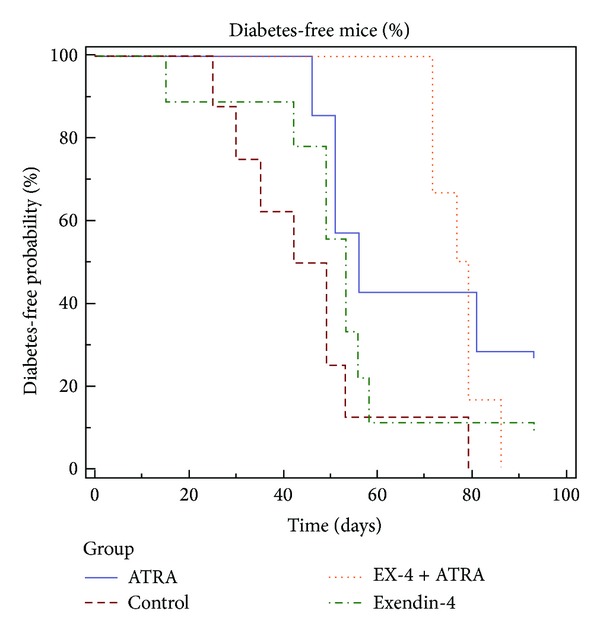
Kaplan-Meier curve of the percentage of diabetes-free mice over time in vehicle (brown line), ATRA- (blue line), exendin-4- (EX-4, green line), and ATRA plus exendin-4- (orange line) treated groups. Adoptive-transfer was performed and treatment was started at day 0. Compared with vehicle, ATRA (*P* = 0.022) and ATRA plus exendin-4 (*P* = 0.013) treatment delayed the onset of diabetes.

**Figure 2:**
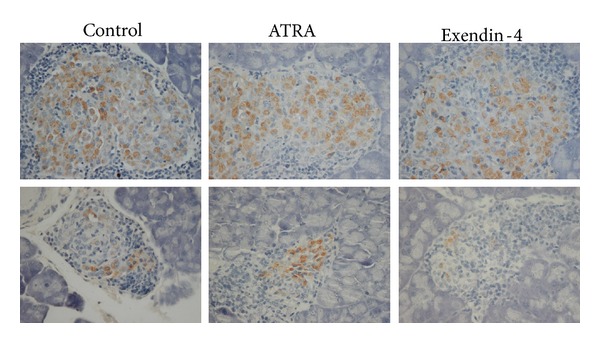
Varied degrees of insulitis with abundant (upper panel) to scanty (lower panel) beta cells were observed in the pancreases of adoptive transferred NOD/scid mice treated with vehicle, ATRA, and exendin-4.

**Figure 3:**
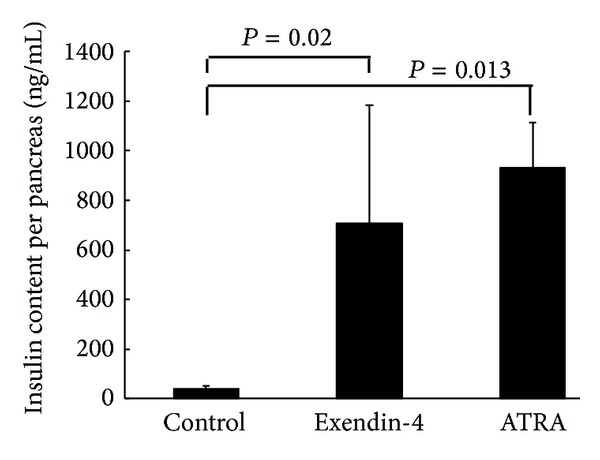
The pancreatic insulin content in adoptive transferred NOD/scid mice treated with vehicle, ATRA, and exendin-4.

**Figure 4:**
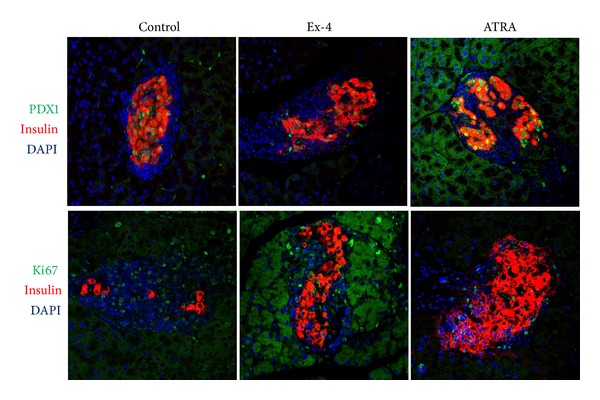
Insulin, Pdx-1, and Ki67 staining for islets in the pancreases of adoptive transferred NOD/scid mice treated with vehicle, ATRA, and exendin-4 (Ex-4).

After adoptive transfer, the mean diabetes-free time in NOD/scid mice treated with vehicle (*n* = 8), ATRA (*n* = 7), exendin-4 (*n* = 9), and ATRA plus exendin-4 (*n* = 6) was 6.5, 8.0, 7.6, and 11.2 weeks, respectively. Compared with vehicle, ATRA (*P* = 0.022) and ATRA plus exendin-4 (*P* = 0.013) treatment delayed the onset of diabetes (Figure 1). Although mice with different treatments had varied degrees of insulitis and a variable number of beta cells per islet (Figure 2), the pancreatic insulin content in mice treated with ATRA (932 ± 182 ng/mL, *n* = 3, *P* = 0.013) and exendin-4 (705 ± 479 ng/mL, *n* = 5, *P* < 0.02) was significantly higher than that of control mice (41 ± 9 ng/mL, *n* = 4) (Figure 3). Moreover, ATRA treated group had more Pdx-1- and Ki67-positive beta cells compared with control and Ex-4-treated animals (Figure 4).

### 3.2. Reversal Study

**Figure 5:**
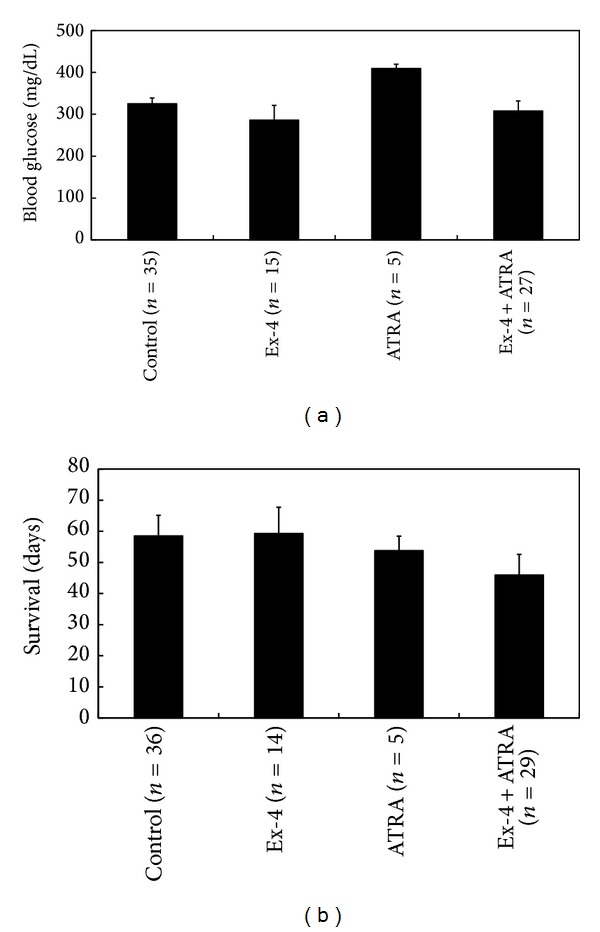
The lowest blood glucose levels (a) and survival time (b) in spontaneous diabetic NOD mice treated with and without ATRA and exendin-4 (Ex-4).

**Figure 6:**
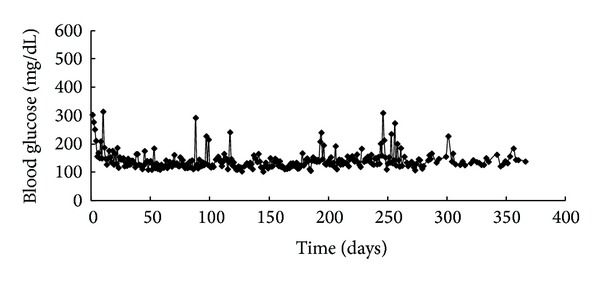
One spontaneous diabetic NOD mouse achieved normoglycemia 10 days after ATRA treatment but then had occasional hyperglycemia between 88 and 366 days.

All but one spontaneous diabetic NOD mouse remained persistent hyperglycemic. The lowest blood glucose level in control (*n* = 35) and mice treated with ATRA (*n* = 5), exendin-4 (*n* = 15), and ATRA plus exendin-4 (*n* = 27) was 325 ± 83, 410 ± 22, 286 ± 136, and 308 ± 125 mg/dL, respectively (*P* = 0.157, Figure 5(a)). Meanwhile, ATRA and/or exendin-4 treatment did not alter their survival time [56 ± 39, 45 ± 19, 59 ± 33, and 46 ± 36 days in control (*n* = 40) and mice treated with ATRA (*n* = 16), exendin-4 (*n* = 24), and ATRA plus exendin-4 (*n* = 29), respectively; *P* = 0.369, Figure 5(b)]. One mouse achieved normoglycemia 10 days after ATRA treatment and then had occasional hyperglycemia between 88 and 366 days (Figure 6).

## 4. Discussion

In the present study, we tested if ATRA and exendin-4 treatment could prevent and reverse autoimmune diabetes. In the prevention experiment, due to limited space in our animal facility, NOD/scid mice were adoptive transferred with splenocytes isolated from 12-week-old female instead of new-onset diabetic NOD mice presented by Van et al. [5]. Even though 100% of our control NOD/scid mice developed diabetes with the mean onset of 6.5 weeks after adoptive transfer [12], in contrast, those treated with ATRA and ATRA plus exendin-4 developed diabetes with the mean onset of 8.0 and 11.2 weeks, respectively. Clearly, ATRA and ATRA plus exendin-4, but not exendin-4, treatment delayed the onset of autoimmune diabetes. This finding indicates that before the onset of diabetes, the inhibition of autoimmunity (exerted by ATRA) is more important than the promotion of beta-cell regeneration (exerted by exendin-4). Since there were varied degrees of insulitis and a variable number of beta cells per islet in the pancreas of mice before death with different treatment, we compared their pancreatic insulin content, an indicator of pancreatic beta-cell mass. Higher pancreatic insulin content was observed in mice treated with ATRA and exendin-4 than in control mice after adoptive transfer. It supports the beneficial effect of ATRA and exendin-4 treatment on beta-cell preservation before the onset of autoimmune diabetes. Our finding of more Pdx-1- and Ki67-positive beta cells in ATRA-treated group compared with control and Ex-4-treated animals indicating that ATRA has replicating and differentiating effects on pancreatic beta cells.

In the reversal experiment, we used spontaneous diabetic NOD mice instead of NOD/scid mice with adoptive transfer. In this slowly progressive diabetic model [13], residual beta cells in the pancreas could be rescued. Unfortunately, nearly all diabetic NOD mice remained persistent hyperglycemic regardless of ATRA and/or exendin-4 treatment. Meanwhile, ATRA and/or exendin-4 treatment did not improve their survival time. Therefore, after the onset of autoimmune diabetes, ATRA cannot halt autoimmune beta-cell destruction. There was only one mouse that achieved normoglycemia after ATRA treatment. It needs to be further studied.

## 5. Conclusions

Before the onset of autoimmune diabetes, ATRA and exendin-4 treatment alone preserves pancreatic beta cells; ATRA and ATRA plus exendin-4 treatment delays the onset of autoimmune diabetes. After the onset of autoimmune diabetes, ATRA and/or exendin-4 treatment is unable to reverse hyperglycemia or improve survival.
